# Antibody-based therapies for emerging infectious diseases.

**DOI:** 10.3201/eid0203.960306

**Published:** 1996

**Authors:** A Casadevall

**Affiliations:** Albert Einstein College of Medicine, Bronx, New York 10461, USA. casadeva@aecom.yu.edu

## Abstract

In the 19th century, it was discovered that immune sera were useful in treating infectious diseases. Serum therapy was largely abandoned in the 1940s because of the toxicity associated with the administration of heterologous sera and the introduction of effective antimicrobial chemotherapy. Recent advances in the technology of monoclonal antibody production provide the means to generate human antibody reagents and reintroduce antibody therapies, while avoiding the toxicities associated with serum therapy. Because of the versatility of antibodies, antibody-based therapies could, in theory, be developed against any existing pathogen. The advantages of antibody-based therapies include versatility, low toxicity, pathogen specificity, enhancement of immune function, and favorable pharmacokinetics; the disadvantages include high cost, limited usefulness against mixed infections, and the need for early and precise microbiologic diagnosis. The potential of antibodies as antiinfective agents has not been fully tapped. Antibody-based therapies constitute a potentially useful option against newly emergent pathogens.


### Antibody-Based Therapies for Emerging Infectious Diseases

**Arturo Casadevall, M.D., Ph.D.**
Albert Einstein College of Medicine, Bronx, New York, USA

In the 19th century, it was discovered that immune sera were useful in treating infectious diseases. Serum therapy was largely abandoned in the 1940s because of the toxicity associated with the administration of heterologous sera and the introduction of effective antimicrobial chemotherapy. Recent advances in the technology of monoclonal antibody production provide the means to generate human antibody reagents and reintroduce antibody therapies, while avoiding the toxicities associated with serum therapy. Because of the versatility of antibodies, antibody-based therapies could, in theory, be developed against any existing pathogen. The advantages of antibody-based therapies include versatility, low toxicity, pathogen specificity, enhancement of immune function, and favorable pharmacokinetics; the disadvantages include high cost, limited usefulness against mixed infections, and the need for early and precise microbiologic diagnosis. The potential of antibodies as antiinfective agents has not been fully tapped. Antibody-based therapies constitute a potentially useful option against newly emergent pathogens.

In the mid-1990s, successful implementation of antiinfective therapy has become increasingly difficult because of widespread antimicrobial resistance, the emergence of new pathogens, and the occurrence of many infections in immunocompromised patients in whom antimicrobial drugs are less effective. Infections caused by some new pathogens (e.g., human immunodefficiency virus [HIV] and *Cryptosporidium parvum*) cannot be cured with existing antimicrobial drugs. Regaining the upper hand in the struggle against microbes requires multidisciplinary efforts which include developing new antimicrobial agents (1), improving surveillance for emerging microbial threats (2), teaching the correct use of antimicrobial therapy (3), expanding the use of vaccines to prevent infection (4), developing adjunctive immunotherapies (5), and conducting new basic research on the mechanisms of pathogenesis and drug resistance. In this article, the potential of antibody therapy in confronting the threat of emergent infections will be explored.

### Antibody-Based Therapies: Then and Now

Antibody-based (serum) therapies were first used to treat human infections in the 1890s (6,7). In the early 20th century, serum therapy was used to treat a variety of bacterial infections, including those cased by *Corynebacterium diphtheriae*, *Strep-*

Address for correspondence: Arturo Casadevall, M.D., Ph.D., Department of Medicine, Albert Einstein College of Medicine, 1300 Morris Park Ave., Bronx, New York 10461, USA; fax: 718-340-8968; e-mail: casadeva@aecom.yu.edu.

**SERUM TREATMENT**
***of Pneumonia***

*Until recently the use of an unconcentrated serum for type I infections represented the only serum treatment for pneumonia which had gained general recognition. While this serum did not affect Type II, Type III or Group IV cases, it proved to be a very effective therapeutic agent in Type I cases in which it was used intravenously in large doses.*

*The obvious difficulties attendant upon the use of large doses of unconcentrated anti-pneumococcus serum have been greatly reduced, Felton and other having succeeded in evolving not only an effective highly concentrated Type I serum but also a corresponding Type II serum. This achievement is of very real significance, since Type I or Type II pneumococci are the causative agents in over fifty per cent of all cases of lobar pneumonia.*

*Promising results have been obtained from the intravenous use of concentrated anti-pneumococcus sera prepared in the Connaught Laboratories, and supplies of these sera are now being made available in four containers as follows:*

**5 cc. & 10 cc. Concentrated Anti-Pneumococcus Serum (Type I)**
**5 cc. & 10 cc. Concentrated Anti-Pneumococcus Serum (Type II)**

*Should there be occasion to administer serum prior to receipt of a report of the typing of a case, a physician may mix these sera.*

*Prices and information regarding the use of Type I and Type II concentrated Anti-Pneumococcus Sera will be gladly supplied upon request.*

**CONNAUGHT LABORATORIES**
**University of Toronto**
**TORONTO 5 • CANADA**

Figure 1. Advertisement for type-specific anti-pneumococcal sera from the March 1931 issue of the *Canadian Medical Association Journal.* The text in this advertisement describes advancements in the preparation of antibody solutions and emphasizes the need for using type-specific serum in the therapy of pneumococcal pneumonia. Note the suggestion that type-specific serum can be mixed for empiric therapy of pneumonia. (Reprinted with permission.)

Reprinted from *Emerging Infectious Diseases*
Vol. 2, No. 3, 200-208, July-September 1996

Table 1. Serum therapy, human MAbs, and antimicrobial chemotherpy

| | Antibody therapy | | Chemotherapy | Comment |
|---|---|---|---|---|
| | Immune serum | Human MAb | | |
| Specificity | Narrow | Narrow | Broad | Narrow specificity avoids selection of resistant organisms among nontargeted microbes. Narrow specificity requires a precise diagnosis before use. |
| Source | Animals<br>Humans | Tissue culture<br>Bioreactor<br>Fermentation | Fermentation<br>Chemical synthesis | Before antibiotics, most serum preparations were from horses and rabbits. MAbs are produced by tissue culture techniques. Industrial production of MAbs may utilize immunoglobulin synthesis in yeast, bacteria, or plants. |
| Toxicity | High | Low | Low | Toxicity of serum was due to allergic reactions to animal protein. Human immunoglobulin preparations are well tolerated (42). Antiidiotypic responses remain a problem for humanized MAb therapy (54). |
| Cost | High | High | Low | Serum therapy for pneumococcal pneumonia in the 1930s was costly(6) Immunoglobulin therapy remains very expensive. |
| Administration | Difficult | Easy | Easy | Serum therapy required considerable expertise, and because of life-threatening allergic reactions, dosage was often based on clinical experience. |
| Pharmacokinetics | Variable | Consistent | Consistent | Pharmacokinetics of heterologous polyclonal antibody depends on multiple variables, e.g.,animal source, isotype composition,and immune status of the recipient. Human MAbs are homogeneous reagents and can be expected to have more consistent pharmacokinetics. |
| Mechanism of action | Antimicrobial<br>Immune enhancement<br>Toxin neutralization | Antimicrobial<br>Immune enhancement<br>Toxin neutralization | Antimicrobial | Conventional antimicrobial chemotherapy kills or inhibits the replication of microorganisms. Antibodies function through a variety of mechanisms, e.g., promoting complement-mediated lysis, enhancing antimicrobial efficacy of host effector cells, efficacy of host effector cells, promoting phagocytosis,preventing attachment, and neutralizing toxins. |

*tococcus pneumoniae*, *Neisseria meningitides*, *Haemophilus influenzae*, group A streptococcus, and *Clostridium tetani* (6,7). By the 1930s, serum therapy was standard treatment for lobar pneumonia (Figure 1). Several large controlled trials showed that administering type-specific serum reduced the death rate in patients with pneumococcal pneumonia by approximately 50% (6). However, when antimicrobial chemotherapy was discovered in the mid-1930s, serum therapy for bacterial infections was rapidly abandoned. Antimicrobial chemotherapy had important advantages over serum therapy: it was more effective and less toxic. The immediate side effects of serum therapy included fevers, chills, and allergic reactions (8,9). A delayed toxic reaction of serum therapy was "serum sickness," a syndrome characterized by rash, proteinuria, and arthralgia; this occurred in 10% to 50% of patients who received heterologous serum and was probably caused by immune complexes. Other disadvantages of serum therapy included the need to establish a precise diagnosis before selecting serum, lot-to-lot variation of serum, and the need for considerable physician expertise (Table 1). Serum therapy could fail because of inadequate dosage, delayed treatment, mis-labeling of serum, and because the infection was mixed or complicated (i.e., empyema) (10). Producing therapeutic sera was very expensive because of the costs of animal husbandry, antibody purification, refrigeration, and standardization by the mouse protection tests. When antimicrobial chemotherapy was first introduced, enthusiasm was expressed for combining serum therapy and antimicrobial chemotherapy. Support for combined therapy came from animal studies, which suggested that combination therapy was more effective than either therapy alone against several pathogens, including group A streptococcus (11), pneumococcus (12), and meningococcus (13), and some authorities recommended combined therapy for serious infections (14,15). However, several studies showed that combined therapy was not more effective than antimicrobial chemotherapy alone and that it caused significantly more side effects (16-18). Therefore, serum therapy was abandoned because it offered no measurable advantage in efficacy over chemotherapy and had substantial disadvantages in implementation, cost, and toxicity.

Today antibody therapy is indicated in infectious diseases in relatively few situations, including replacement therapy in immunoglobulin-deficient patients, post-exposure prophylaxis against several viruses (e.g., rabies, measles, hepatitis A and B, varicella), and toxin neutralization (diphtheria, tetanus, and botulism). Ironically, the general abandonment of antibodies as antimicrobial agents was followed by major advances in the technology of antibody production. In 1975, hybridoma technology provided the means to generate unlimited amounts of monoclonal antibodies (MAbs) (19). In recent years, major advances have been made in the techniques used to generate human antibodies and humanize murine MAbs (20).

The juxtaposition of three recent developments makes the reintroduction of antibody-based therapies an option for serious consideration. First, because of advances in technology, human antibody reagents can be synthesized; thus the toxicities traditionally associated with serum therapy can be avoided. Second, the emergence of new pathogens, the reemergence of old pathogens, and the increased prevalence of drug-resistant microorganisms have caused the effectiveness of existing therapeutic options to decline. Third, the difficulties involved in treating infections in immunocompromised patients suggest the need for adjunctive immunotherapy.

### Polyclonal Sera Versus Monoclonal Antibodies for Therapy

Immune sera contain antibodies of multiple specificities and isotypes. Problems with immune sera include lot-to-lot variation (21), low content of specific antibodies (22), and some hazards in the transmission of infectious diseases (23). Commercially available intravenous immunoglobulin preparations obtained from human donors differ in their opsonic activity for common pathogens such as *Staphylococcus epidermidis*, *H. influenzae* type b, *S. pneumoniae*, group B streptococcus, and *Escherichia coli*, reflecting the characteristics of the donor pool (22). In contrast MAbs are generated in vitro by either hybridoma technology or recombinant DNA techniques. MAbs are homogenous immunoglobulins that, by definition, recognize one epitope and have markedly higher specific activity than polyclonal preparations. For example, 0.7 mg of two human MAbs to tetanus toxin have the same activity as 100 to 170 mg of immune globulin (24). The higher specific activity of MAbs may also translate into greater therapeutic efficacy. MAbs formulations are superior to polyclonal sera in homogeneity, constancy, specific activity, and (possibly) safety. For some infections,polyclonal preparations may be superior to MAbs because MAbs contain antibodies to multiple epitopes (i.e., they are polyvalent). However, different therapeutic MAbs can be combined to generate polyvalent preparations composed of antibodies with multiple specificities and isotypes. Given the advantages of MAb preparations over immunesera, antibody-based therapies for emergent infections, if used, will likely rely primarily on MAb technology.

### Advantages of Antibody-Based Therapies

Humans can produce antibodies to practically all existing pathogens. Antibody molecules are assembled from combinations of variable gene elements, and the possibilities resulting from combining the many variable gene elements in the germline enable the host to synthesize antibodies to an extraordinarily large number of antigens. During the generation of the antibody response, somatic mutations are introduced into immunoglobulin genes, which result in higher affinity antibodies and more diversity in specificity (25). Thus, antibodies are, as a class, broad-spectrum antimicrobial agents with activity against all classes of pathogens. However, individual antibodies are usually pathogen-specific. Pathogen-specific antimicrobial agents have the theoretical advantage that they do not select for resistant organisms among nontargeted microbes and are unlikely to produce great disturbances in the normal host flora.

Antibody-based therapies could, in theory, be developed against any pathogen. Although the level of antibody immunity differs among pathogens, it may be possible to develop useful antibody therapies even if natural antibody immunity plays little or no role in protection. Two fungi, *Candida albicans* and *Cryptococcus neoformans*, are pathogens for which protective antibodies can be generated despite uncertainty about the role of natural antibody immunity (26). The MAbs to *C. neoformans* enhance the therapeutic efficacy of amphotericin b (27,28), fluconazole (29), and 5-flucytosine (30) in murine models of cryptococcosis. Therefore, uncertainty regarding the role of natural antibody immunity in protecting against a given pathogen does not rule out the existence of antibodies that may be useful in therapy.

Microbial targets for therapeutic antibody development are not necessarily limited to extracellular pathogens. Although intracellular pathogens are commonly believed to be outside the reach of antibody immunity, several reports have suggested that some MAbs are active against some intracellular microorganisms. Some IgA MAbs can neutralize intracellular viruses (31), and an MAb to *Toxoplasma gondii* has been reported to interfere with intracellular replication of the parasite (32). It has been proposed that intracellular virus neutralization by IgA occurs by antibodies binding to viral proteins and interfering with viral assembly (31). Additional evidence for intracellular antibody activity comes from the observation that IgG anti-DNA autoantibodies can enter the cytoplasm and nucleus of living cells (33).

Antibodies mediate antimicrobial function through a variety of mechanisms, including inhibition of microbial attachment, agglutination, viral neutralization, toxin neutralization, antibody-directed cellular cytotoxicity, complement activation, and opsonization (34). Antibodies are extremely versatile antimicrobial molecules: some are active directly against the pathogen, some neutralize the toxic products of infection, and others enhance the efficacy of host effector cells. Some MAbs to poliovirus are neutralizing only at fever temperatures (35), which demonstrates their ability to function at physiologic extremes. The versatility of antibody-based therapies is illustrated by the ability of digoxin-binding antibodies to reverse digoxin toxicity (36) and recent attempts to treat septic shock by employing MAbs that bind cytokines (37).

Human IgG has favorable pharmacokinetics for use as an antimicrobial agent, including good tissue penetration (38) and a half-life of about 20 days (39). Murine MAbs have much shorter half-lives in humans, and these usually elicit human antibody responses (40). Human-mouse chimeric antibodies and humanized MAbs are synthetic molecules composed primarily of human antibody protein sequences that retain the antigen-binding site of the heterologous antibody (20). Human-mouse chimeric antibodies and humanized MAbs have longer half-lives than the murine precursor, but their half-lives are still much shorter than that of native human IgG (41). This area is being intensively investigated, and genetic engineering of antibody molecules may be used to synthesize MAbs with longer half-lives.

Immunoglobulin therapy with human reagents is generally well tolerated (42). Serious adverse reactions, including renal failure (43), aseptic meningitis (44), and thromboembolic events (45) can occur with high-dose (0.5 to 2 g/kg) antibody therapy. However, antiinfective immunoglobulin therapy with MAb preparations is unlikely to require the high doses of immunoglobulin used to treat rheumatic disorders and other conditions. For example, the heterologous immune sera used in the preantibiotic era were effective, although they contained small amounts of specific antibody. The higher activity of MAb preparations should permit a smaller amount of immunoglobulin proteins to be used and thus avoid the occasional toxicity reported with high-dose antibody therapy.

### Problems with Antibody-Based Therapies

Most antibody therapies are pathogen-specific. This is a disadvantage in dealing with mixed infections. Mixed infection with multiple *S. pneumoniae* serotypes was recognized as a cause for the failure of serum therapy (46). For pathogens that are antigenically variable, one solution is to use antibody cocktails of agents active against the most common antigenic types. Antibody cocktails may also be designed to include antibodies of different isotypes to enhance antibody effector function. The successful implementation of antibody-based therapies would also require improvements in diagnostic microbiology. In the preantibiotic era, for lobar pneumonia, rapid protocols were developed for the isolation and typing of pneumococci from sputum (47). Recent advances in diagnostic microbiology, including polymerase chain reaction and nucleic acid hybridization, could substantially shorten the time required to establish a microbiologic diagnosis. The narrow spectrum of antimicrobial activity that characterizes antibody-based therapies is a drawback for commercial development, however. Pathogen-specific drugs, have smaller potential markets than broad-spectrum antimicrobial agents, and this makes them less attractive to the pharmaceutical industry. Conversely, the emergence of multidrug-resistant microorganisms and new pathogens for which no drugs exist could make pathogen-specific drugs attractive for commercial development.

Widespread use of antibody-based therapies could produce selective pressure on microbial populations for the emergence of antibody-resistant variants. Antibody-resistant mutants of *Borrelia burdorferi* have been produced in the laboratory (48), and may be selected in patients who undergo antibody-based therapies. Microorganisms may become resistant to antibodies by acquiring mutations that change the antigenic site recognized by the anti-bodies or by producing proteases that destroy immunoglobulins. The IgA protease genes of *N. gonorrhoeae* can be transferred between strains, and the widespread use of antibody therapies may select for protease-producing strains (49). However, the versatility of antibody technologies provides alternatives for countering antibody-resistant strains. For instance, new antibodies directed toward the mutated epitope could be developed, or antibodies that bind other antigenic targets could be introduced. Protease-producing strains could be countered with protease-resistant immunoglobulin molecules generated by introducing amino acid changes at protease cleavage sites. Alternatively, MAbs that neutralize proteases could be incorporated into therapeutic antibody cocktails in a manner analogous to the present practice of using beta-lactamase inhibitors to increase the effectiveness of beta-lactam antibiotics. Recognizing that the introduction of new agents has been inevitably followed by the emergence of resistance, researchers could attempt to minimize the emergence of antibody-resistant organisms from the outset by using cocktails of MAbs directed at multiple antigenic targets.Combining antibody therapy with chemotherapy also could reduce the probability of selecting for organisms resistant to either therapeutic modality.

Antibodies are more effective in preventing infection than in treating established infection. Antibody-based therapies have been most useful when administered early in the course of disease: serum therapy for pneumococcal pneumonia was most effective if serum was administered within 3 days of the onset of clinical symptoms (6). Because antibodies are proteins, therapy for invasive infections is likely to require systemic administration. This is a serious disadvantage in developing countries where access to medical care is limited. For enteric pathogens, oral antibody administration may be most effective (50,51). Efforts to develop MAbs for cancer therapy have run into unexpected pharmacologic problems (38,40). The finding that MAb uptake by tumors is partially dependent on antigen expression in the tumor (40) suggests that MAb uptake by infected tissues could depend on microbial antigen expression at the site. The same problems may be found when antibody penetrates tumors.

Antibodies can, in principle, elicit neutralizing antibody, allergic responses, or both. Administering rodent MAbs to patients elicits human antibodies to the rodent MAb (52). Antibody therapies against emergent pathogens, if attempted, are likely to use human, human-animal chimeric, or humanized antibodies. Mouse-human chimerics and humanized antibodies are less immunogenic than heterologous antibodies (41,53,54); therefore, the likelihood that the patient will mount a neutralizing antibody response to the therapeutic antibody may be reduced. Nevertheless, antiidiotypic responses have been observed in patients receiving humanized antibody therapy (54). The clinical importance of such antiidiotypic responses is uncertain. Many infections are single life-threatening episodes in the life of a person, and the occurrence of antiidiotypic antibodies following therapy may require repeated or long-term antibody administration.

Antibodies are largely excluded from the central

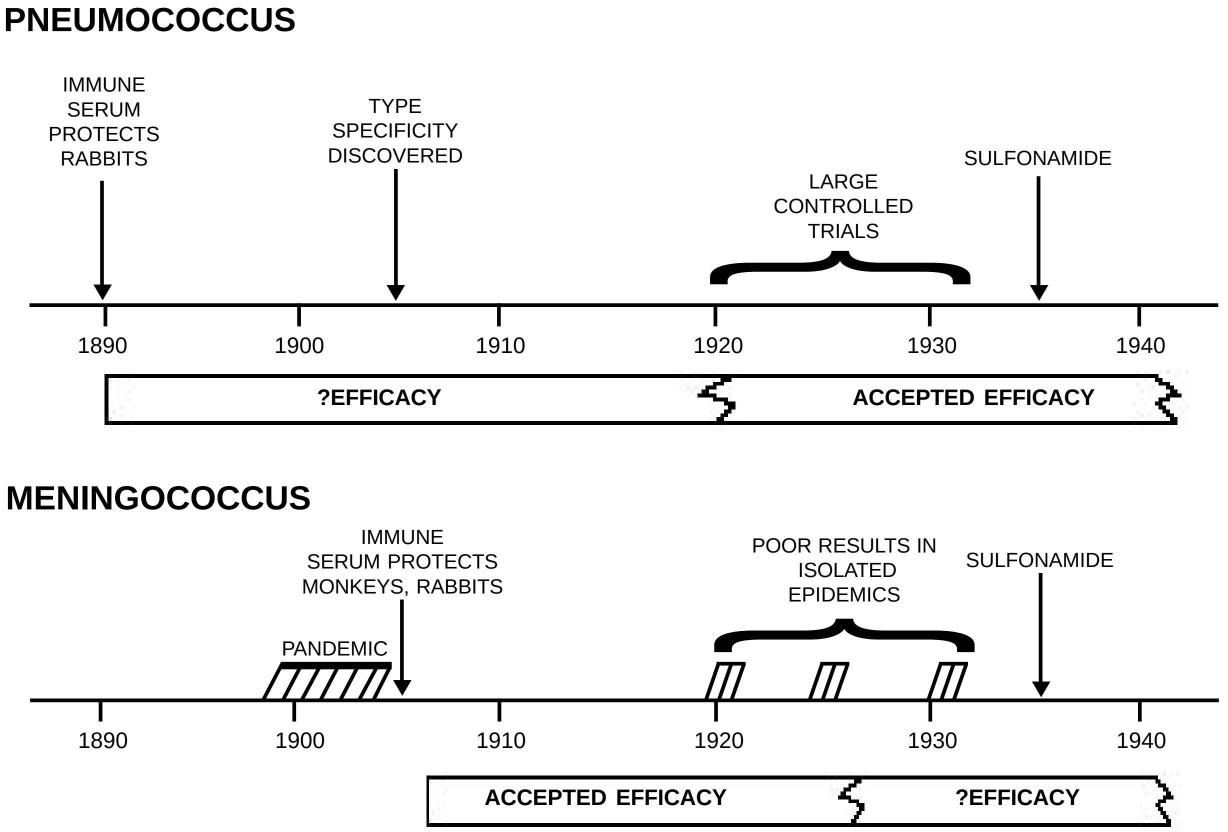

Figure 2. Schematic illustration of the major events in the development of serum therapy for pneumococcal pneumonia and meningococcal meningitis. For pneumococcal pneumonia, considerable uncertainty existed regarding the usefulness of serum therapy in the decades following the demonstration that immune sera could transfer protection to animals. However, the discovery that type-specific serum necessary for efficacy, followed by extensive clinical trials, led to the general acceptance of serum therapy for pneumococcal pneumonia in the late 1920s. For meningococcal meningitis, the antisera generated against the strains prevalent in the early 1900s proved to be effective in therapy. However, the efficacy of serum therapy in later epidemics of meningococcal meningitis was significantly lower, leading to uncertainty about the value of serum therapy for this infection (16).

nervous system by the blood-brain barrier. Nevertheless, antibody treatment of brain infections is feasible. In some brain infections, the blood-brain barrier is more permeable to serum components because of inflammation, and systemic antibody therapy was used successfully to meningococcal meningitis (55). If antibody penetration to brain tissue is a problem, two alternatives exist. First, antibodies can be administered directly into the subarachnoid space (as was done for the treatment of meningococcal meningitis in the preantibiotic era) (15,56). Second, antibody molecules can be engineered for enhanced brain penetration by altering their charge (57) or by linking them to proteins that cross the blood-brain barrier (58).

Antibody therapies are also costly to develop and expensive for the patient. For example, antibody prophylaxis for cytomegalovirus infections can cost several thousand dollars per patient (59). To be cost-effective, antibody-based therapies would have to provide a clear benefit over existing therapy. For emerging pathogens for which no therapy is available, the cost of antibody therapies may be justifiable, depending on the potential for death, illness, and long-term consequences of the infection. In the long run, advances in antibody production and improvements in technology may greatly lower costs and make antibody-based therapy more competitive with antimicrobial chemotherapy.

### Historical Perspective on the Development of Antibody Therapies

In recent years, considerable interest has been expressed in using antibody-based therapies to treat septic shock (60). Unfortunately, MAbs to endotoxin have not been as effective in clinical trials as anticipated (61,62), and this has dampened some of the enthusiasm for MAb therapies. The development of serum therapy for *S. pneumoniae* also encountered considerable difficulties in the preantibiotic era.

The Klemperers demonstrated that immune serum protected rabbits in 1891 (63), but reliable antibody therapies for pneumococcal pneumonia were not available until the 1920s (Figure 2). Translating the laboratory finding that immune sera protected rabbits against experimental pneumococcal infection to the successful use of serum therapy for lobar pneumonia in humans required extensive basic and clinical research. At the laboratory bench, developing antibody therapy for pneumococcal pneumonia required the discovery that antigenic variation existed among pneumococcal strains, that only type-specific sera provided protection, that certain vaccination schedules were necessary to elicit good antibody responses, the ability to standardize the serum potency (by the mouse protection test), and improved antibody purification techniques (6). At the bedside, implementing successful serum therapy required learning when and how to give serum, managing the side effects of serum therapy, and developing rapid protocols for recovering pneumococci from sputum for serum typing (64). The mouse protection test reduced but did not eliminate the problems of lot-to-lot variation in serum efficacy (21). The development and perfection of antibody therapies for pneumococcus contributed important research findings, which led to major discoveries in immunology (65). The high death rate for meningococcal meningitis also led to the rapid development of serum therapy (6). In the early 1900s, serum therapy markedly reduced the death rate of meningococcal epidemics, possibly because of antigenic changes in the pathogen (6). The lengthy time required for the development of serum therapy for pneumococcus, the variable efficacy of antimeningococcal sera (depending on the epidemic), and the more recent difficulties encountered in developing MAb therapy for septic shock suggest that developing antibody-based therapies for emergent pathogens will require extensive preclinical and clinical testing.

### Other Antibody-Based Strategies Against Emerging Pathogens

Vaccines that elicit protective antibody immunity could be used to protect against emergent pathogens. A polysaccharide-protein conjugate vaccine is being developed (66) against *C. neoformans*. This vaccine elicits protective antibodies in mice (67), and it is hoped that vaccination will result in the production of effective anti-cryptococcal antibodies to prevent disease in patients at risk. The conjugate vaccine against *C. neoformans* is intended to elicit protective antibody immunity, even though the role of natural antibody immunity in protection against cryptococcosis is uncertain (26). Newer vaccines against common pathogens could help limit the spread of drug-resistant microorganisms. Dissemination of penicillin-resistant pneumococci has been associated with infection and carriage by young children among whom the current 23-valent pneumococcal polysaccharide vaccine is ineffective in inducing protective immunity (4). However, the effectiveness of polysaccharide-protein conjugate vaccine to *H. influenzae* type b suggests that a similar conjugate vaccine to *S. pneumoniae*, if available, could effectively abort childhood infection with antibiotic-resistant pneumococci and thereby limit the spread of these strains (4).

### Future Directions

Immunoglobulins are an extremely versatile class of antimicrobial proteins that can be used to prevent and treat emerging infectious diseases. Antibody therapy has been effective against a variety of diverse microorganisms. The historical record clearly documents both the usefulness and the difficulties in developing and implementing passive antibody therapies. The experience with serum therapy for pneumococcal pneumonia and meningococcal meningitis suggests that extensive basic and clinical research is essential for the successful implementation of antibody therapy. Given the multitude of pathogens, the pathogen-specific nature of antibody therapies, and the costs of developing and using antibody therapies, the development of such therapies for most pathogens at present, would be impractical. However, for selected pathogens, antibody-based therapies could provide new therapeutic options. Opportunities for the development of antibody-based strategies include 1) pathogens for which there is no available antimicrobial therapy (e.g., *C. parvum* and vancomycin-resistant enterococcus); 2) pathogens that affect primarily immuno-compromised patients in whom antimicrobial therapy is not very effective (e.g., invasive fungal infections); 3) pathogens for which drug-resistant variants are rapidly spreading (e.g., *Pseudomonas aeruginosa* [68]); and 4) highly virulent pathogens for which few effective antimicrobial agents are available (e.g., methicillin-resistant *S. aureus*).

### Acknowledgments

The author thanks Drs. L. Pirofski, M.D. Scharff, and R. Soeiro for many useful discussions over the years. This research was supported in part by NIH grants R01-AI33774 and R01-AI13342.

*Dr. Casadevall is assistant professor of medicine and microbiology and immunology at the Einstein College of Medicine. His research interests include antibody immunity, fungal pathogenesis, and molecular epidemiology.*

### References

1. Service RF. Antibiotics that resist resistance. Science 1995;270:724-7.
2. Berkelman RL, Bryan RT, Osterholm MT, LeDuc JW, Hughes JM. Infectious disease surveillance: a crumbling foundation. Science 1994;264:368-70.
3. Joshi N, Milfred D. The use and misuse of new antibiotics. Arch Intern Med 1995;155:569-77.
4. Mumford RS, Murphy TV. Antimicrobial resistance in *Streptococcus pneumoniae*: can immunization prevent its spread. J Invest Med 1994;42:613-21.
5. Roilides E, Pizzo PA. Modulation of host defenses by cytokines: evolving adjuncts in prevention and treatment of serious infections in immunocompromised hosts. Clin Infect Dis 1992;15:508-24.
6. Casadevall A, Scharff MD. "Serum therapy" revisited: animal models of infection and the development of passive antibody therapy. AntimicrobAgents Chemother 1994;38:1695-702.
7. Casadevall A, Scharff MD. Return to the past: the case for antibody-based therapies in infectious diseases. Clin Infect Dis 1995;21:150-61.
8. Rackemann FM. Allergy: serum reactions, with particular reference to the prevention and treatment of tetanus. JAMA 1942;226:726-33.
9. Feinberg SM. The therapy of (horse) serum reactions. general rules in the administration of therapeutic serums. JAMA 1936;107:1717-9.
10. Finland M. The use of serum, sulfanilamide, and sulfapyridine in the treatment of pneumococcic infections. Med Clin North Am 1939;1205-9.
11. Colebrook L, Maxted WR. Streptococcal infections in mice treated by chemotherapy and serum. Lancet 1940;1:21-8.
12. MacLeod CM. Chemotherapy of pneumococcic pneumonia. JAMA 1939;113:1405-10.
13. Branham SE. Sulphanilamide, serum, and combined drug and serum therapy in experimental meningococcus and pneumococcus infections in mice. Public Health Rep 1938;52:685-95.
14. Sako W, Dwan PF, Platou ES. Sulfanilamide and serum in the treatment and prophylaxis of scarlet fever. JAMA 1938;111:995-7.
15. Alexander HE. Treatment of *Haemophilus influenzae* infections and of meningococcic and pneumococcic meningitis. Am J Dis Child 1943;66:172-87.
16. Waghelstein JM. Sulfanilamide in the treatment of 106 patients with meningococcic infections. JAMA 1938;111:2172-4.
17. Dowling HF, Abernethy TJ. The treatment of pneumococcus pneumonia: a comparison of the results obtained with specific serum and sulfapyridine. Am J Med Sci 1940;199:55-62.
18. Cory CW, Abbot CE, Truszkowski EG. Treatment of meningococcic meningitis and septicemia. J Pediatr 1944;25:35-48.
19. Kohler G, Milstein C. Continuous cultures of fused cells secreting antibody of predefined specificity. Nature 1975;256:495-7.
20. Wright A, Shin S-U, Morrison SL. Genetically engineered antibodies: progress and prospects. Crit Rev Immunol 1992;12:125-68.
21. Felton, LD. The units of protective antibody in anti-pneumococcus serum and antibody solution. J Infect Dis 1928;43:531-42.
22. Weisman LE, Cruess DF, Fischer GW. Opsonic activity ofcommercially available standard intravenous immunoglobulin preparations. Pediatr Infect Dis J 1994;13:1122-5.
23. Slade HB. Human immunoglobulins for intravenous use and hepatitis C viral transmission. Clin Diagn Lab Immunol 1994;1:613-9.
24. Lang AB, Cryz SJ, Schurch U, Ganss MT, Bruderer U. Immunotherapy with human monoclonal antibodies. J Immunol 1993;151:466-72.
25. French DL, Laskov R, Scharff MD. The role of somatic hypermutation in the generation of antibody diversity. Science 1989;244:1152-7.
26. Casadevall A. Antibody immunity and invasive fungal infections. Infect Immun 1995;63:4211-8.
27. Mukherjee J, Zuckier L, Scharff MD, Casadevall A. Therapeutic efficacy of monoclonal antibodies to *Cryptococcus neoformans* glucuronoxylomannan alone and in combination with amphotericin B. Antimicrob Agents Chemother 1994;38:580-7.
28. Dromer F, Charreire J. Improved amphotericin B activity by a monoclonal anti-*Cryptococcus neoformans* antibody: study during murine cryptococcosis and mechanisms of action. J Infect Dis 1991;163:1114-20.
29. Mukherjee J, Feldmesser M, Scharff MD, Casadevall A. Monoclonal antibodies to *Cryptococcus neoformans* glucuronoxylomannan enhance fluconazole activity. Antimicrob Agents Chemother 1995;39:1398-405.
30. Feldmesser M, Mukherjee J, CasadevallA. Combination of 5-flucytosine and capsule binding monoclonal antibody in therapy of murine *Cryptococcus neoformans* infections and in vitro. J Antimicrob Chemother. 1996; (in press).
31. Mazanec MB, Kaetzel CS, Lamm ME, Fletcher D, Nedrud JG. Intracellular neutralization of virus by immunoglobulin A antibodies. Proc Natl Acad Sci 1992;89:6901-5.
32. Mineo JR, Khan IA, Kasper LH. *Toxoplasma gondii*: A monoclonal antibody that inhibits intracellular replication. Experimental Parasitol 1994;79:351-61.
33. Yanase K, Smith RM, Cizman B, Foster MH, Peahy LD, Jarret L, et al. A subgroup of murine monoclonal anti-deoxyribonucleic acid antibodies traverse the cytoplasm and enter the nucleous in a time and temperature-dependent manner. Lab Invest 1994;71:52-60.
34. Heinzel FP. Antibodies. In: Mandell GL, Bennett JE, Dolin R, editors. Principles and practice of infectious diseases New York: Churchill Livingstone, 1995:36-57.

35. Delaet I, Boeye A. Monoclonal antibodies that disrupt poliovirus only at fever temperatures. J Virol 1993;67:5299-302.
36. Smith TW, Butler VP, Haber E, Fozzard H, Marcus FI, Bremmer WF., et al. Treatment of life-threatening digitalis intoxication with digoxin-specific Fab antibody fragments. N Engl J Med 1982;307:1357-62.
37. Bobmer M, Fournel MA, Hinshaw LB. Preclinical review of anti-tumor necrosis factor monoclonal antibodies. Crit Care Med 1993;21:S441-6.
38. Zuckier LS, Rodrigues LD, Scharff MD. Immunologic and pharmacologic concepts of monoclonal antibodies. Semin Nucl Med 1989;19:166-86.
39. Waldman TA, Strober W. Metabolism of immunoglobulins. Prog Allergy 1969;14:1-110.
40. Reilly RM, Sandhu J, Alvarez-Diez TM, Gallinger S, Kirsh J, Stern H. Problems of delivery of monoclonal antibodies: pharmaceutical and pharmacokinetic solutions. Clin Phamacokinet 1995;28:126-42.
41. LoBuglio AF, Wheeler RH, Trang J, Haynes A, Rogers K, Harvey EB, et al. Mouse/human chimeric monoclonal antibody in man:kinetics and immune response. Proc Natl Acad Sci USA 1989;86:4220-4.
42. Pennington JE. Newer uses of intravenous immunoglobulins as anti-infective agents. Antimicrob Agents Chemother 1990;34:1463-6.
43. Pasatiempo AMG, Kroser JA, Rudnick M, Hoffman BI. Acute renal failure after intravenous immunoglobulin therapy. J Rheumatol 1994;21:347-9.
44. Sekul EA, Cupler EJ, Dalakas MC. Aseptic meningitis associated with high-dose intravenous immunoglobulin therapy: frequency and risk factors. Ann Intern Med 1994;121:259-62.
45. Wolff SN, Fay JW, Herzig RH, Greer JP, Dummer S, Brown RA, et al. High-dose weekly intravenous immunoglobulin to prevent infections in patients undergoing autologous bone marrow transplantation or severe myelosuppressive therapy. Ann Intern Med 1993;118:937-42.
46. Bullowa JGM. The management of the pneumonias. New York:Oxford University Press, 1937.
47. Bullowa JGM. The reliability of sputum typing and its relation to serum therapy. JAMA 1935;105:1512-8.
48. SadzieneA, Rosa PA, Thompson PA, Hogan DM, Barbour AG. Antibody-resistant mutants of *Borrelia burgdorferi*: In vitro selection and characterization. J Exp Med. 1992;176:799-809.
49. Halter R, Pohlner J, Meyer TF. Mossaic-like organization of IgA protease genes in *Neisseria gonorrhoeae* generated by horizontal genetic exchange in vivo. EMBO J 1989;8:2737-44.
50. Barnes GL, Doyle LW, Hewson PH, et al. A randomised trial of oral gammaglobulin in low-birth-weight infants infected with rotavirus. Lancet 1982;1:1371-3.
51. Borowitz SM, Saulsbury FT. Treatment of chronic cryptosporidial infection with orally admininstered human serum immune globulin. J Pediatr 1991;119:593-5.
52. Schroff RW, Foon KA, Beatty SM, Oldham RK, Morgan AC. Human anti-murine immunoglobulin responses in patients receiving monoclonal antibody therapy. Cancer Res 1985;45:879-85.
53. Lazarovits AI, Rochon J, Banks L, Hollomby DJ, Muirhead N, Jevnikar AM, et al. Human mouse chimeric CD7 monoclonal antibody (SDZCHH380) for the prophylaxis of kidney transplant rejection. J Immunol 1993;150:5163-74.
54. Issacs JD, Watts RA, Hazleman BL, Hale G, Keogan MT, Cobbold SP, et al. Humanized monoclonal antibody therapy for rheumatoid arthritis. Lancet 1992;340:748-52.
55. Hoyne AL. Intravenous treatment of meningococcic meningitis with meningococcus antitoxin. JAMA 1936;107:478-81.
56. Flexner S. The results of the serum treatment in thirteen hundred cases of epidemic meningitis. J Exp Med 1913;17:553.
57. Triguro D, Buciak JB, Yang J, Pardridge WM. Blood-brain barrier transport of cationized immunoglobulin G: enhanced delivery compared to native protein. Proc Natl Acad Sci 1989;86:4761-5.
58. Friden PM, Walus LR, Musso GF, Taylor MA, Malfroy B, Starzyk RM. Anti-transferrin receptor antibody and antibody-drug complexes cross the blood-brain barrier. Proc Natl Acad Sci 1991;88:4771-5.
59. Conti DJ, Freed BM, Gruber SA, Lempert N. Prophylaxis of primary cytomegalovirus disease in renal transplant recipients. Arch Surg 1994;129:443-7.
60. Wolff SM. Monoclonal antibodies and the treatment of gram-negative bacteremia and shock. N Engl J Med 1991;324:486-7.
61. Fink MP. Adoptive immunotherapy of gram-negative sepsis:use of monoclonal antibodies to lipopolysaccharide. Crit Care Med 1994;21:S32-9.
62. Loewenthal L, Berlin MD. Combined serum and sulphanilamide in the treatment of streptococcal infections in mice. Lancet 1939;1:197-9.
63. Klemperer G and Klemperer F. Versuche uber immunisirung und heilung bei der pneumokokken-infection. Berl Klin Wochenschr 1891;28:833-5.
64. Bullowa JGM. Serum Therapy. In: The management of the pneumonias, New York: Oxford University Press, 1937:283-362.
65. Watson DA, Musher DM, Jacobson JW, Verhoef J. A brief history of the pneumococcus in biomedical research: a panoply of scientific discovery. Clin Infect Dis 1993;17:913-24.
66. Devi SJN, Schneerson R, Egan W, Ulrich TJ, Bryla D, Robbins JB, et al. *Cryptococcus neoformans* serotype A glucuronoxylomannan-protein conjugate vaccines: synthesis, characterization, and immunogenicity. Infect Immun 1991;59:3700-7.
67. Mukherjee J, Scharff MD, Casadevall A. Protective murine monoclonal antibodies to *Cryptococcus neoformans*. Infect Immun 1992;60:4534-41.
68. Pier GB, Thomas D, Small G, Siadak A, Zweerink H. In vitro and in vivo activity of polyclonal and monoclonal human immunoglobulins G, M, and A against *Pseudomonas aeroginosa* lipopolysaccharide. Infect Immun 1989;57:174-9.
